# Thermal Conductivity of Composite Materials Copper-Fullerene Soot

**DOI:** 10.3390/ma15041415

**Published:** 2022-02-14

**Authors:** Tatiana Koltsova, Elizaveta Bobrynina, Aleksei Vozniakovskii, Tatiana Larionova, Olga Klimova-Korsmik

**Affiliations:** 1 Institute of Machinery, Materials, and Transport, Peter the Great St. Petersburg Polytechnic University, 195251 Saint Petersburg, Russia; bobrynina@inbox.ru (E.B.); larionova@hotmail.com (T.L.); 2 World-Class Research Center “Advanced Digital Technologies”, Saint Petersburg State Marine Technical University, 190121 Saint Petersburg, Russia; o.klimova@ltc.ru; 3 Laboratory “Physics for Cluster Structures”, Ioffe Institute of Russian Academy of Sciences, 194021 Saint Petersburg, Russia; alexey_inform@mail.ru

**Keywords:** composite, powder metallurgy, thermal conductivity, fullerene soot

## Abstract

Copper-based composites strengthened with fullerene soot nanoparticles of 20–30 nm size in concentration up to 23 vol.% were prepared via two methods: mechanical mixing and molecular level mixing. The dependence of thermal conductivity on the carbon concentration was studied. Maxwell’s model describes well the change in the thermal conductivity of the composite obtained by molecular level mixing. However, thermal conductivity of the composite produced by mechanical mixing is significantly lower than the calculated values, due to structural inhomogeneity and residual stresses. Comparison of the thermal conductivity of Cu-fullerene soot composites with that of Cu-based composites described in the literature showed that the prepared materials are not inferior in thermal conductivity to composites containing carbon nanotubes, despite the fact that fullerene soot has a much lower thermal conductivity.

## 1. Introduction

Copper is a common and economical material with high conductivity, but at the same time pure copper is considered a low strength material. Alloying allows the obtainment of copper compounds with high mechanical characteristics, but significantly reduces the conductivity of copper. Dispersion hardening can create a material with high strength and little loss of conductivity. Carbon nanotubes and fullerenes can significantly increase the mechanical properties of a material. Scientists are interested in adding nanocarbon into a metal matrix; for example, carbon nanotubes (CNTs) have extremely high thermal conductivity (about 3000 Wm^−1^K^−1^ [1]). With the addition of CNTs into a copper matrix, an increase in strength characteristics and conductivity is expected, but in fact, the overall thermal conductivity of copper-nanocarbon materials can even be reduced due to random orientation of CNTs, high interfacial resistance at the copper-CNT interface, porosity, agglomeration of nanostructures and other negative factors [2,3,4,5]. In this regard, the method of adding carbon nanostructures and the method of compaction of the composite material significantly affect the thermal conductivity of composites.

An increase in thermal conductivity with the addition of multi-walled CNTs (MWCNTs) up to 1.5 vol.% into a copper matrix was reported in [6]. Experimental data of the thermal conductivity of composites Cu with 0–1.5 vol.% MWCNTs agree with the analytical assessment for composites reinforced by randomly oriented fibers [7,8]. Previously, the authors carried out various chemical treatments of MWCNTs to remove the amorphous component from the surface of nanotubes [2,6]. Composite materials were obtained by wet mechanical mixing of CNTs and copper particles, followed by compaction by spark plasma sintering (SPS). The relative density of the material varied from 96.8–99.0%. The maximum value of thermal conductivity was obtained at 1 vol.% and amounted to ~359 Wm^−1^K^−1^. With an increase in the content of CNTs, the authors attribute the decrease in thermal conductivity to an increase in the number of copper-CNT contacts and a thermal barrier at the phase interface.

In other works, with the addition of CNTs by mechanical mixing, an increase in thermal conductivity was not achieved. In [3] for composites obtained by mechanical mixing and hot pressing, the thermal conductivity of the material practically did not change with the carbon content and was 320 Wm^−1^K^−1^ at 5 vol.% and 10 vol.% CNTs, but sharply decreased to 245 Wm^−1^K^−1^ with the addition of 15 vol.% CNTs.

The relative density of the composites resulted in 96.1–98.4%. The authors note that no increase in thermal conductivity was observed, due to the thermal resistance of the interface and the random orientation of the tubes. At high CNT contents, a decrease in thermal conductivity is associated with the clustering of nanotubes.

In [4], composite materials with MWCNTs were obtained by mechanical mixing in ethanol followed by reduction in hydrogen. Compaction was carried out in several stages: cold pressing with sintering and cold rolling with annealing. The measurement results showed that the best thermal conductivity of the Cu-CNT composite was ~300 Wm^−1^K^−1^, which is lower than that of pure copper at the same compaction modes (~355 Wm^−1^K^−1^). The article notes the positive effect of annealing of composites caused by the recrystallization of copper grains. However, even after annealing, the values remained below the thermal conductivity of similar samples of pure copper. The decrease in thermal conductivity is explained by thermal resistance at the CNT-copper interface. Nonetheless, it can be assumed that the compaction method did not allow the obtainment of a high density of the samples, and therefore the values of thermal conductivity were lower than the conductivity of pure copper. The density values of the samples are not indicated in the work.

In work [5], Cu and Cu-1 vol.% CNTs samples with densities of 99.21% and 97.04%, respectively, were obtained via ultrasonic mixing of copper and CNTs in ethanol followed by consolidation by the SPS method. Thermal conductivity of the Cu-1vol.% CNTs composite was 273 Wm^−1^K^−1^, and that of the Cu sample was 362 Wm^−1^K^−1^. In addition to porosity and a thermal barrier at the interface, the authors note a high oxygen content in the sample, which negatively affects the thermal properties.

A significant increase in thermal conductivity upon the addition of CNTs was reported in [9]. The thermal conductivity of composites containing 0.25 wt.% (1.23 vol.%) CNTs with a diameter of 20–40 nm reached 364.5 Wm^−1^K^−1^at a relative density of 90.9%, while the conductivity of pure copper was determined as 274.3 Wm^−1^K^−1^ at a density of 83.8%. The composites were obtained by molecular blending of CNTs with copper (II) acetate monohydrate and compacted by cold pressing followed by microwave sintering. The study of thermal conductivity was carried out depending on the diameter of CNTs, as well as their grain size and sample density. The authors note that the density of the composite material has the greatest effect on thermal conductivity. Molecular level mixing produces materials with less stress and less oxide, but residual porosity can make the effect less noticeable.

In previous works [10,11], we showed the possibility of obtaining composite materials of the Cu-fullerene soot (FS) system with physical and mechanical characteristics at the level of copper-CNTs materials. Fullerene soot is a mixture of fullerenes with amorphous carbon and has a lower cost compared to pure fullerenes and CNTs. However, the thermal conductivity of fullerenes and fullerene soot is significantly lower than the thermal conductivity of the copper matrix. This article discusses materials obtained by two different methods: mechanical mixing and molecular level mixing. The results are compared with copper-CNTs materials.

## 2. Materials and Methods

**Figure 1:**
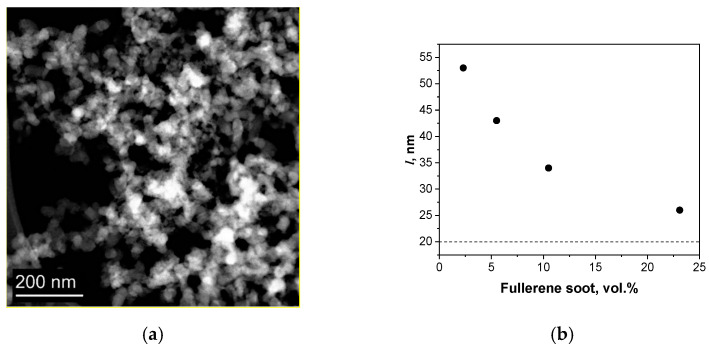
TEM image of fullerene soot (**a**), average distance (*l*) between strengthening particles in Cu-FS composite (**b**).

To prepare Cu-fullerene soot composites, fullerene soot produced by Suzhou Dade Carbon Nanotechnology Co (Suzhou, China) was used (Figure 1). Two different methods for composite powder preparation were used: mechanical milling (MM) and molecular level mixing (MLM).

To obtain composite powder via mechanical milling, commercial copper powder of PMS-1 grade (GOST 4960–2009, HimSnab, Saint Petersburg, Russia) of 99.5% purity produced by the electrolytic method was used. The Cu particles were dendrite crystals with an average size of 40 µm. Cu-FS powder of assigned FS concentration was produced in a planetary mill Fritsch Pulverisette 7 premium line ball mill (FRITSCH, Markt Einersheim, Germany), with steel balls in argon at 200 rpm and 600 rpm; a detailed description of the process is given in [10].

To prepare composite powder via molecular level mixing, first, FS suspension of assigned concentration was prepared in a copper nitrate solution (FS was precoated with sodium lauryl sulfate in order to improve the carbon wettability with a solution), then, the suspension was sprayed with compressed air on a Spray Dryer B-290 (BUCHI, Flawil, Switzerland) at a temperature of 150–200 °C, with a solution feed rate of 5 mL/min. Further, the resulting powder was annealed at 400 °C in an oxidizing atmosphere for 60–120 min to obtain a CuO-FS powder, and then the oxide was reduced to copper in a hydrogen atmosphere at 550 °C for 120 min [11]. For the reference, the samples of pure copper without additives were prepared by the two considered methods using described modes.

The samples were compacted by cold pressing at a pressure of 400 MPa, followed by hot pressing at 750 °C and a pressure of 200 MPa [10,11]. The density of the composites was determined by Archimedes’ principle. For the calculation of the theoretical density of the composites, the density of pure copper and fullerene soot were taken as 8.92 g/cm^3^ and 1.56 g/cm^3^ [12], respectively. Fullerene soot observation was carried out using a Transmission Electron microscopy Talos F200X (Thermo Fisher Scientific, Waltham, MA, USA). For optical observation, the specimens were prepared through a conventional mechanical polishing and then were etched in mixed FeCl_3_/HCl solution. The optical microscopy was carried out on a Carl Zeiss Observer D1m microscope (ZEISS, Oberkochen, Germany). Scanning electron microscopy (SEM) images were obtained on MIRA 3 TESCAN microscope (Tescan, Brno-Kohoutovice, Czech Republic); Brinell hardness was tested with ZWICK ZHU (ZwickRoell, Ulm, Germany) equipment under a load of 100 N and dwelling time of 10 s.

Relative density was calculated as the ratio of the real density of the sample to its theoretically estimated density. The real density was measured by hydrostatic weighting after the samples had been coated with wax. GH-202 scale with AD-1653 (A&D Engineering, Tokyo, Japan) kit was used. The real density was determined according to

$$\rho =\frac{M_{1}\rho_{w}\rho_{p}}{\left(M_{2}-M_{3}\right)\rho_{p}-M_{4}\rho_{w}}$$

where: M_1_ = mass of the sample without protective layer, g; M_2_ = mass of the sample with protective layer suspended in air, g; M_3_ = mass of the sample with protective layer suspended in liquid, g; M_4_ = mass of the protective layer, g; *ρ_w_* = water density, g/mm^2^; and *ρ_p_* = density of the protective layer, g/mm^2^. Theoretically estimated densities were calculated using the mixture rule, taking into account the assigned volume fraction of the FS. The density of pure copper and fullerene soot were taken as 8.92 g/cm^3^ and 1.57 g/cm^3^ [12], respectively.

The measurements of thermal diffusivity (α) were carried out by the flash method on the DXF-200 equipment (TA-Instruments, New Castle, DE, USA). To create a pulse of energy on the front side of the sample, the xenon flash lamp was used, and the change in temperature on the back side of the sample was fixed by contact thermocouple. The duration of the flash was 500 ms. The specific heat capacity (C_p_) was determined via a comparison method using flash analysis as well. The reference and tested samples were measured under the exact same conditions, following which the specific heat capacity was determined as

$$C_{p}=C_{r}\cdot \left(m_{r}\cdot \Delta T_{r}\right)/\left(m_{s}\cdot \Delta T_{s}\right)$$

where $C_{r}$ is the specific heat capacity of the reference sample, J/(kg·K); $m_{r}$ is the mass of the reference sample (kg); $m_{s}$ is the mass of the test sample (kg); $\Delta T_{r}$ is the temperature change of the reference sample (K); and $\Delta T_{s}$ is the temperature change of the test sample (K). Thermal conductivity was calculated according to

*k* = α·ρ·C_*p*_

where α—thermal diffusivity (m^2^/s); ρ—density (kg/cm^3^); and C_*p*_—specific heat capacity (J·kg^−1^·K^−1^) [13].

## 3. Results and Discussion

For the study, Cu-FS composite powder was prepared in two ways: by mechanical mixing and molecular level mixing. The prepared composites had carbon concentrations of 2.3; 5.5; 10.5; and 23.1 vol.% fullerene soot, which corresponded to 0.5; 1.0; 2.0; and 5.0 wt.%. The relative density of the samples prepared via MM was 90.5–96.0%, and that of the samples prepared by MLM was 97.0–98.5%. The relative density and hardness of the composites are presented in Table 1.

The fullerene soot consists of spherical particles with an average size of d = 20 nm (Figure 1a). The average distance (l) between the particles was calculated according to the equation $l=d\cdot \sqrt[{3}]{\left(\pi /(6\cdot \varphi )\right)}$, where *φ* is the volume fraction of the dispersed particles; it was assumed that the particles were separate from each other and uniformly distributed in the matrix.

**Figure 2:**
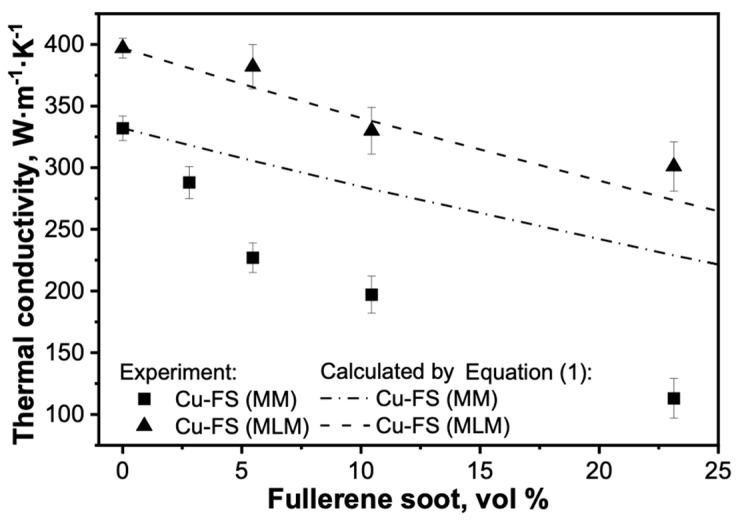
Thermal conductivities of Cu-FS composites at 25 °C as a function of FS content.

At the considered concentrations, the average distance between the centers of the nanoparticles exceeds the particle diameter (20 nm, indicated by the dotted line in Figure 1b). In this regard, the fullerene soot can be considered as isolated particles in a copper matrix without interaction between them. In this case, the thermal conductivity can be described by the Maxwell model [14], which is used to estimate the thermal conductivity in mixtures and composites. For a two-component mixture, which is represented by distributed spherical particles, Maxwell’s model gives the following heat equation:

(1) $$\frac{k_{eff}}{k_{m}}=1+\frac{3\cdot \varphi }{\left(\frac{k_{1}+2\cdot k_{m}}{k_{1}-k_{m}}\right)-\varphi }$$

where *k_eff_* is the thermal conductivity of the composite material (Wm^−1^K^−1^), *k_m_* is the thermal conductivity of the matrix (Wm^−1^K^−1^), *k_1_* is the thermal conductivity of the filler particles (Wm^−1^K^−1^), and *φ* is the volumetric filling of the composite material. The thermal conductivity of the FS was adopted equal to 0.2 Wm^−1^K^−1^, based on the works [15,16]. The *k_m_* values for each method were determined by measuring the thermal conductivity of the pure copper samples obtained via analogous modes: 397 Wm^−1^K^−1^ for the MLM method and 332 Wm^−1^K^−1^ for the MM method. The results of the calculated and experimental values are shown in Figure 2.

The experimental values for the samples obtained by molecular level mixing are close to the calculated ones. Some values turned out to be higher than the calculated ones, which may be caused by a lower content of FS in real samples, since, possibly, soot could be partially removed from the powder during synthesis at the stages of oxidative or reductive annealing. In general, it is worth noting the high thermal conductivity of the samples prepared by MLM. Up to 10.5 vol.% of FS the thermal conductivity of the composite is higher than that of pure copper subjected to mechanical milling. Thus, molecular level mixing allows the obtainment of powders with high purity and uniform distribution of the carbon particles, and the avoidance of negative factors affecting thermal conductivity, such as high porosity, oxides, stresses, or carbon particles agglomeration.

**Figure 3:**
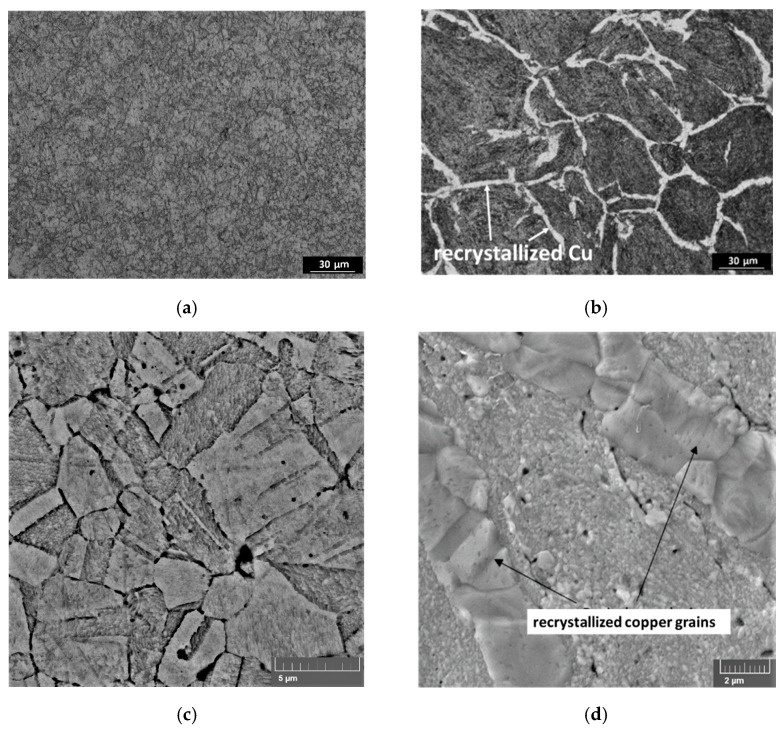
Optical (**a**,**b**) and SEM (**c**,**d**) images of the microstructure of the composite materials Cu-5.5 vol.% FS obtained by molecular level mixing (**a**,**c**) and mechanical milling (**b**,**d**).

For the samples obtained by mechanical milling, the experimental values are lower than the calculated ones, and with an increase in the FS concentration, the difference increases. As we noted earlier [10], the structure of the samples differs significantly depending on the material preparation method. Materials obtained by MLM have a homogeneous and fine-grained structure (Figure 3a,c), while in the composites prepared by mechanical milling, a network consisting of recrystallized copper grains is observed (Figure 3b,d); this phenomenon was discussed in [10]. The recrystallized copper grains should have a positive effect on thermal conductivity, but the positive effect was not observed due to other structural features: the agglomeration of carbon particles, partial oxidation, porosity, and residual stresses.

**Figure 4:**
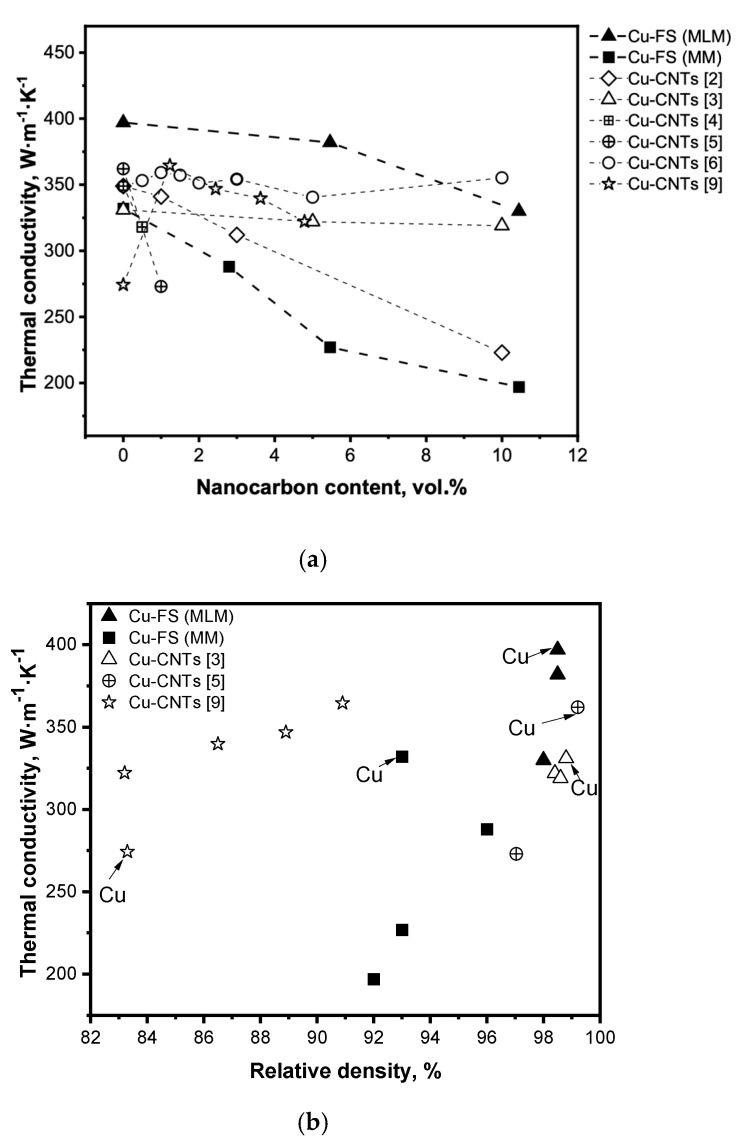
The dependences of thermal conductivity on the nanocarbon content (**a**) and the composites density (**b**) for the Cu-FS composites produced in the present work and for Cu-CNTs composites [3,5,9].

A comparison of the thermal conductivity of Cu-FS composites with that of Cu-CNTs materials discussed in the literature is shown in Figure 4a. As can be seen from the graph, the values of thermal conductivity for the samples with CNT are mainly between the values presented in this study, with different methods of adding fullerene soot. Figure 4b shows the dependence of thermal conductivity on the density of the produced composites as well as literature data on Cu-CNTs materials. In spite of materials of different composition and produced by different methods being considered, certain regularities should be noted. Firstly, there is a strong trend of increasing conductivity with the increase of density; secondly, at a similar density the samples of pure copper have higher conductivity than composites (except [9]). It is obvious that one of the reasons for the high conductivity of the Cu-FS composites obtained by MLM is the high density of the composite.

Comparison of the thermal conductivity of Cu-FS composites with the data described in the literature showed that the obtained materials are not inferior in thermal conductivity to composites containing CNTs. The addition of CNTs into a copper matrix does not lead to a significant increase in thermal conductivity due to the presence of an interphase barrier, porosity, stresses, and oxides. The best results were obtained by the authors at a high density of samples and a low content of CNTs (up to 1.5 vol.%). Although fullerene soot does not have a high thermal conductivity, due to the high density, uniform distribution of carbon in copper, a material with an FC content of up to 5 vol.% has a higher thermal conductivity than that of copper-CNTs materials.

## 4. Conclusions

To summarize, the addition of fullerene soot into the copper matrix leads to a decrease in the thermal conductivity of the material. Maxwell’s model describes well the change in the thermal conductivity of Cu-FS materials obtained by molecular level mixing. This clearly indicates that the method allows the obtainment of a composite of high density and homogeneous structure, and that it may be used for the synthesis of carbon reinforced copper -based composites with excellent physical properties. In the case of mechanical milling, the structure of the material is not uniform and the porosity is more significant; therefore, the thermal conductivity is significantly lower than the calculated values. Comparison of the thermal conductivity of Cu-FS composites obtained by molecular level mixing with the data described in the literature showed that the obtained materials are not inferior in thermal conductivity to composites containing CNTs.

## Figures and Tables

**Table 1:** Properties of the composite.

Fullerene Soot Content, Vol.%	Preparation Method			
	Mechanical Milling		Molecular Level Mixing	
	Hardness, HB	Relative Density, %	Hardness, HB	Relative Density, %
0	75 ± 3.0	93.0 ± 0.5	36 ± 1.8	98.5 ± 0.2
2.3	154 ± 2.2	96.0 ± 0.7	-	-
5.5	141 ± 2.6	93.0 ± 0.5	80 ± 2.3	98.5 ± 0.4
10.5	134 ± 3.1	92.0 ± 0.4	115 ± 3.0	98.0 ± 0.3
23.1	79 ± 2.6	90.5 ± 0.8	127 ± 2.4	97.2 ± 0.3

## Data Availability

Not applicable.
